# Recurrent post-operative endophthalmitis caused by *Sphingomonas paucimobilis* despite vitrectomy – a case and review of the literature

**DOI:** 10.1186/s12348-023-00325-6

**Published:** 2023-03-06

**Authors:** Luke Tran, Rylan Hayes, Andrew Apel, Thomas P Moloney

**Affiliations:** 1 Department of Ophthalmology, Princess Alexandra Hospital, Brisbane, QLD Australia; 2 School of Medicine, University of Queensland, Brisbane, QLD Australia; 3 The Eye Health Centre, Wickham Terrace, Brisbane, QLD Australia; 4 Vitreoretinal Unit, Department of Ophthalmology, Royal Brisbane and Women’s Hospital, Brisbane, QLD Australia

## Abstract

Over the past 20 years, scattered reports have emerged about a low virulence, gram negative bacillus, *Sphingomonas paucimobilis*, causing unpredictable clinical presentations of endophthalmitis. Previous reports have characterised the organism as being resistant to aggressive treatment and prone to recurrence up to several months later, with few warning signs of any residual infection. We report a case of a 75 year-old male who returned 10 days after a left eye cataract surgery with an atypical, indolent endophthalmitis. He was treated with broad-spectrum intravitreal antibiotics and vitrectomy, and despite initial improvement, the patient suffered a recurrence after 2 weeks, necessitating successive rounds of intravitreal antibiotics. While our patient was able to achieve an excellent final visual acuity of 6/9, there are several cases in the literature reporting similar cases with much worse visual outcomes. Further research is required to elucidate early warning signs that may indicate a recurrence of *S. paucimobilis* infection, and the underlying mechanism by which it is resistant to standard endophthalmitis therapy. Alongside this case, we review and summarise the literature on postoperative endophthalmitis involving this organism.

## Introduction

Postoperative endophthalmitis is a rare complication of intraocular surgeries that often culminates in devastating visual outcomes for patients [1]. The identification and investigation of potential causative pathogens is crucial in predicting the clinical course of the disease and ultimately, the best approach to treatment. Among these causative agents, studies have reported that infections with gram negative organisms are often associated with far poorer visual outcomes [2, 3] .

One such gram-negative organism, *Sphingomonas paucimobilis*, has received relatively sparse coverage, with fewer than 10 total case reports in the last 15 years that implicate the organism in a postoperative endophthalmitis [4]. Known for its low virulence, the organism is more often seen in infections of the immunocompromised, although, cases have also been reported in healthy hosts. While some authors report rapid resolution of symptoms and return to baseline visual acuity with appropriate treatment, the organism has also been characterised as a non-responder to aggressive surgical and medical management and thus warrants further research [4]. Here, we report a case of delayed acute onset of postoperative endophthalmitis involving *S. paucimobilis* that recurred despite aggressive treatment. Given the varied clinical courses within these cases, and a lack of literature that ties all these different presentations of postoperative *S. paucimobilis* endophthalmitis together, we also provide a review of the literature on this topic.

## Case report

A 75-year-old man presented to the emergency department 10 days after a routine cataract extraction and implantation of a posterior chamber intraocular lens (IOL) in the left eye, complaining of increased floaters and painless cloudy vision. Postoperative reviews were unremarkable and there were no complications requiring additional procedures or reoperation. There were no symptoms or concerns even at day 7 post-op when the patient returned to have cataract surgery in his right eye. Review of the patient’s medical history was unremarkable with no reported ocular history outside of the aforementioned cataract surgeries. He was not immunocompromised.

**Fig. 1:**
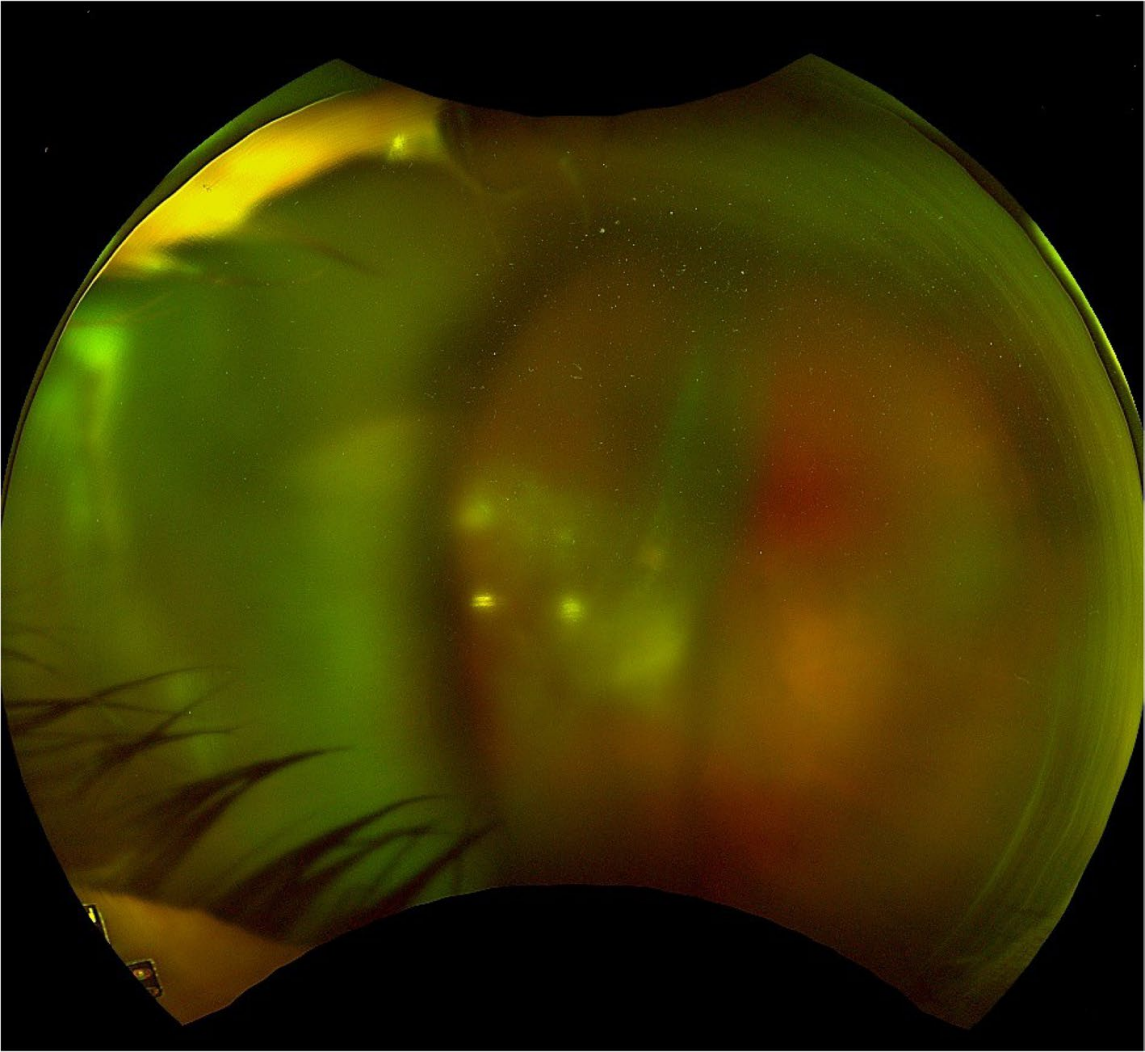
Optos photo of left eye on day of presentation and vitrectomy

**Fig. 2:**
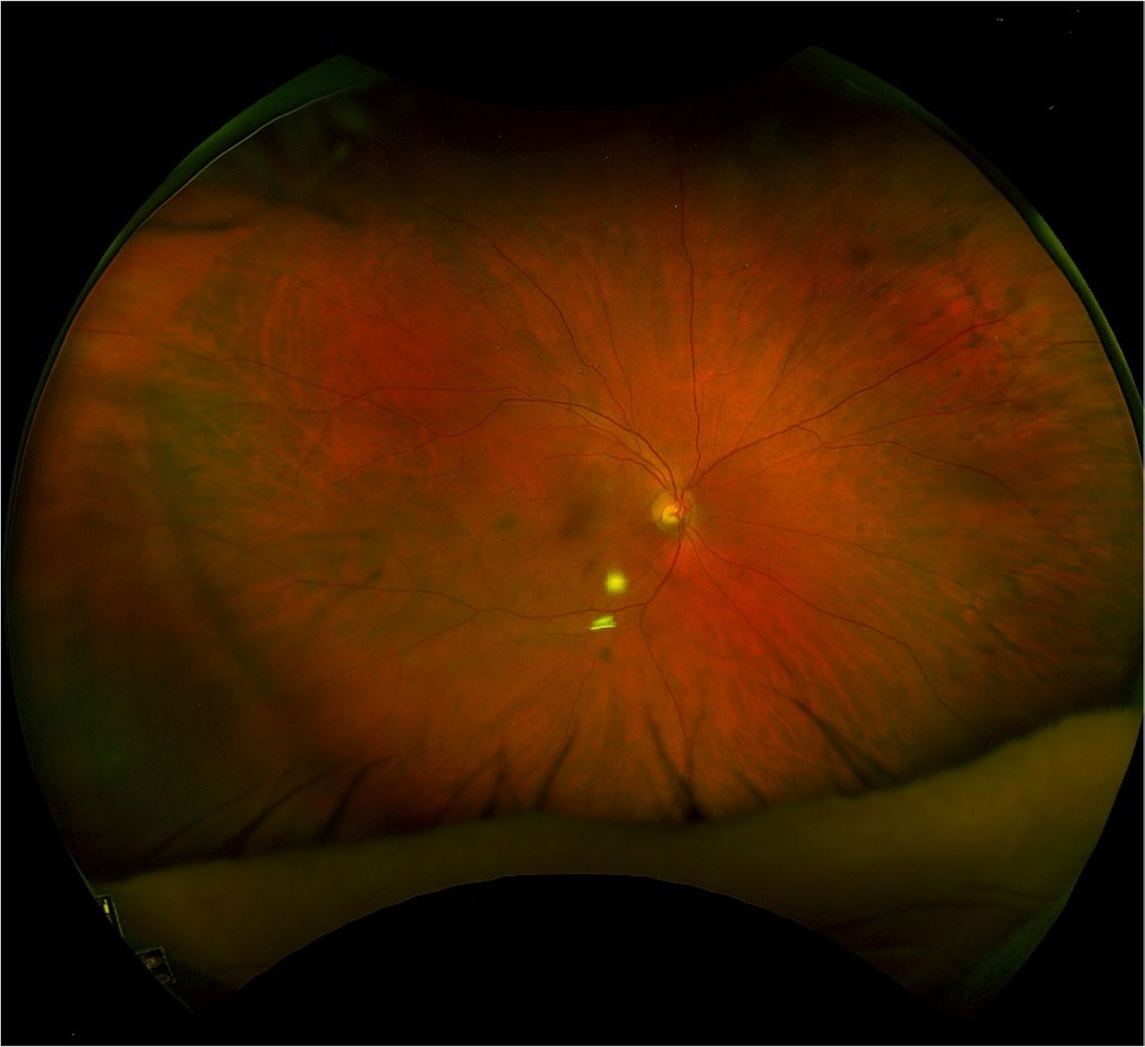
Optos photo of right eye on day of presentation

On initial examination, best corrected visual acuity (BCVA) was 6/9 in the right eye and counting fingers in the left eye. Intraocular pressure (IOP) was 15mmHg in both eyes. Anterior segment examination of the left eye revealed only very mild conjunctival injection, corneal oedema with mild Descemet membrane folds, 2 + anterior chamber cells and a central clear IOL. In the posterior segment, there was extensive vitritis mainly centrally and inferiorly with small focal areas of retinal vasculitis/pallor inferiorly (Fig. 1). Examination and fundal imaging of the right eye was normal (Fig. 2). Despite appearing indolent on examination, the patient’s progressively deteriorating visual acuity was a strong indication to proceed with vitrectomy and intravitreal injection of vancomycin and ceftazidime. The procedure was completed without complication and samples of vitreous humour obtained from the vitrectomy were sent for microscopy, culture, and sensitivities (MCS).

Day 1 post vitrectomy, the patient’s BCVA in the left eye improved to 6/18 − 1 and the patient reported no post operative pain. Provisional MCS results were positive for leucocytes and gram-negative bacilli with cultures pending.

One week after the operation, the patient’s BCVA in the left eye improved to 6/12. Examination revealed 1 + cells in the anterior chamber, an improving view into the posterior segment with mild residual vitritis and an attached retina. It was at this stage that culture isolated *Sphingomonas paucimobilis*, which was sensitive to, ciprofloxacin, cotrimoxazole, ceftazidime, meropenem and piperacillin-tazobactam and resistant to gentamicin and tobramycin. Given the patient’s improvement, they were advised to return in 1 week for ongoing review.

**Fig. 3:**
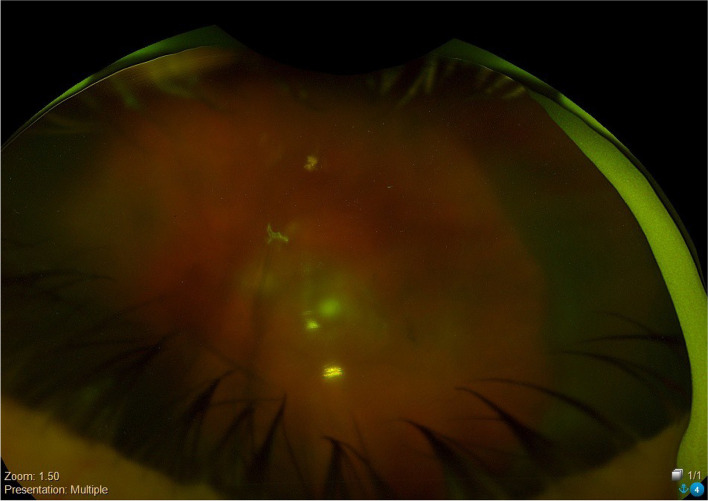
Optos photo of left eye at time of recurrence of endophthalmitis

At the 2 weeks follow up, the patient reported redness and intermittent watery discharge from the left eye. Left eye BCVA had once again deteriorated to counting fingers. Examination showed a 1 mm hypopyon and dilated fundus exam showed recurrence of the severe vitritis (Fig. 3). The patient reported no issues in the right eye with examination in the right eye continuing to be unremarkable. Given the recurrence of the patient’s endophthalmitis, intravitreal injection of ceftazidime was repeated.

**Fig. 4:**
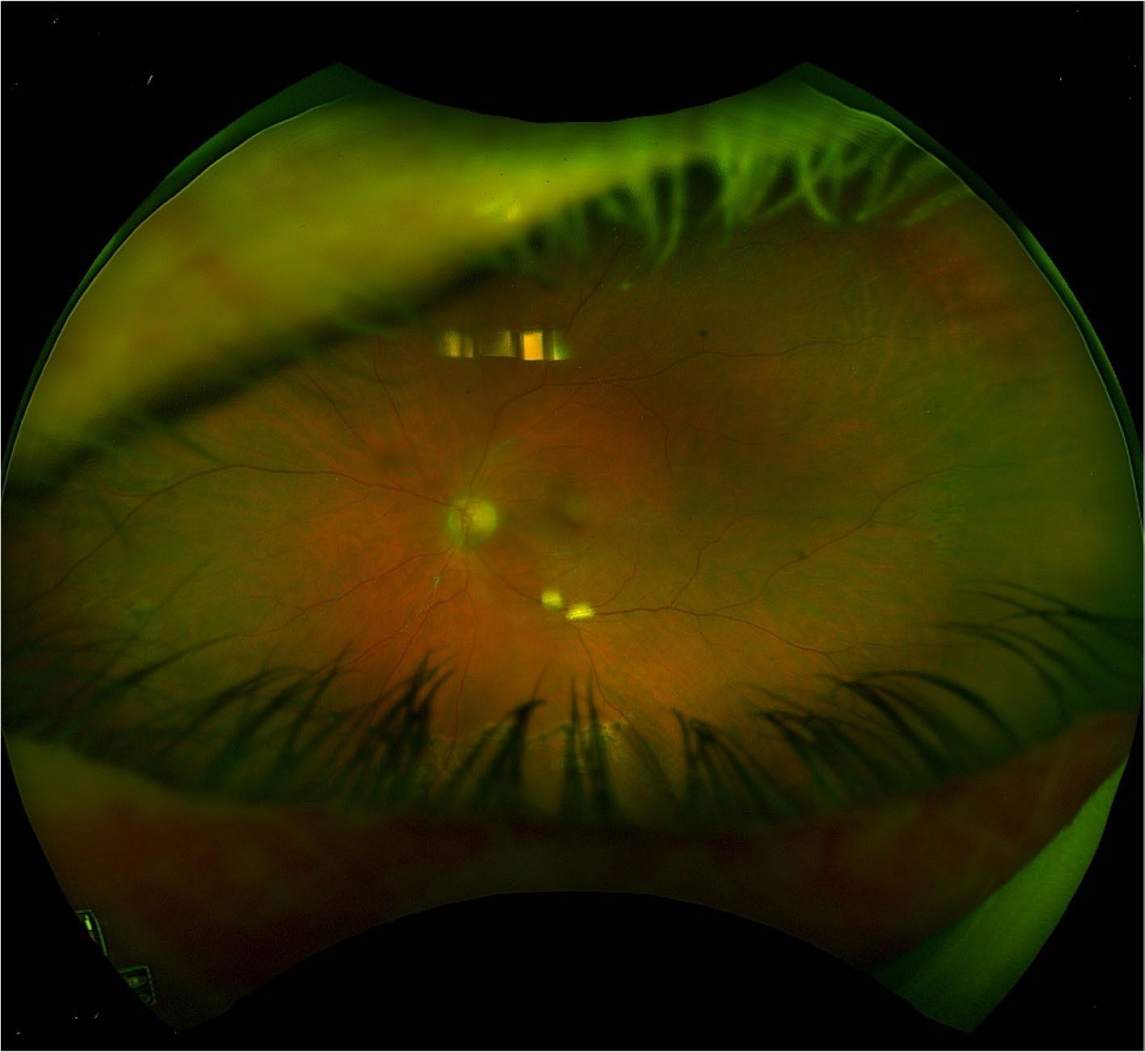
Optos photo of left eye 2 months after initial presentation

At the 3-week mark, the patient’s vision had recovered again to 6/12 in the left eye and examination revealed 0.5 + anterior chamber cells only and improving vitritis. The patient received a final dose of intravitreal ceftazidime and in subsequent follow up appointments over the next month saw complete resolution of the disease. The final uncorrected visual acuity in the left eye was 6/9, with examination revealing a quiet anterior chamber and only mild residual haze in the posterior segment (Fig. 4).

## Discussion

Postoperative endophthalmitis is a rare yet clinically significant disease entity that has been extensively studied in the literature [5]. Amongst all cases of endophthalmitis, cases with gram negative involvement are far less prevalent than their gram-positive counterparts, yet they are often associated with worse prognoses and poorer visual outcomes [2]. To the best of our knowledge, there have been less than 10 confirmed reports of endophthalmitis caused by *Sphingomonas paucimobilis* to date.

First isolated from human specimens in 1977, *Sphingomonas paucimobilis* has traditionally been described in the literature as an opportunistic pathogen, typically associated with cases of meningitis, peritonitis, and septicaemia in the immunocompromised [6, 7]. It’s involvement in ophthalmic disease has been limited further still, with only a few scattered case reports since 2006, describing a mix of endophthalmitis and infectious keratitis [4].

Despite being found widely in the hospital setting, in places such as water distribution systems, respiratory therapy equipment and as a commensal organism in the conjunctival sac, its low virulence has often led to it being regarded as a clinically unimportant entity [7, 8]. This low virulence is suspected to be the underlying mechanism by which patients develop an indolent endophthalmitis that often delays presentation, and how patients can have recurrence that occurs several months after treatment [9]. Somewhat paradoxically however, there are also cases reported of fulminant endophthalmitis occurring within days of surgery that are resistant to treatment and result in extremely poor visual outcomes [4, 10].

The first case of *S. paucimobilis* associated endophthalmitis was described by Adams et al. in 2006. Their patient initially re-presented with endophthalmitis within 24 h of the surgery and was successfully cleared after 3 weeks with aggressive antibiotics and vitrectomy. Despite this, the patient seemingly suffered a recurrence of her endophthalmitis with the same organism 7 months after her initial complication [9]. Kelkar et al. also reported a case with rapid onset, where the patient presented with symptomatic endophthalmitis the day after the surgery but provide no further details in their study [11]. Seo et al. on the other hand, presented a case of delayed post operative endophthalmitis, occurring acutely 3 months after the initial cataract surgery. This case responded well to vitrectomy and antibiotics with no further recurrence [12]. Likewise, Mitra et al. reported a case who developed delayed endophthalmitis 1 month after his surgery, who saw complete resolution of his disease with vitrectomy and an intense antibiotics regimen [13]. Another case has been reported in China, although, the findings remain untranslated [14].

While previous studies have noted some degree of resistance to antibiotics and surgical treatment, Agarwal et al. have reported a case of fulminant *S. paucimobilis* associated endophthalmitis that was completely non-responsive to three rounds of intravitreal antibiotics and vitrectomies. Despite confirming sensitivity to the intravitreal ceftazidime and gentamycin, as well as oral coverage with fortified gentamycin and trimethoprim-sulfamethoxazole, the disease continued to progress and eventually culminated in a visual acuity of no perception of light in the affected eye and phthisis bulbi [4]. Similarly, Garrido et al. observed a case with rapidly evolving disease in a previously immunosuppressed patient. The patient presented 48 h postoperatively with a severe endophthalmitis that continued to progress despite maximal medical therapy, leading to eventual evisceration of the affected eye [10].

In our case, we observed a rare gram-negative bacterium causing an atypical presentation of endophthalmitis that delayed treatment, and also, a recurrence of disease despite aggressive sensitivity-confirmed antibiotics and vitrectomy. To the best of our knowledge, this atypical presentation of early *S. paucimobilis* endophthalmitis with equivocal findings in a white and painless eye, has yet to be described in the literature and is likely due to the low virulence of the organism. Further research into the early warning signs of this rare disease will assist in earlier identification and prompt initiation of treatment, likely leading to better visual outcomes.

While our patient was fortunate enough to have achieved a very good visual outcome in the end, there are multiple cases of ophthalmic infections with *S. paucimobilis* in the literature, where recurrence or resistance to treatment has led to dismal final visual acuity or sometimes even evisceration [4, 10]. Resistance of *S. paucimobilis* to sensitivity-confirmed antibiotics is a common theme, with the degree of resistance ranging from slowed resolution of disease to seemingly complete non-responsiveness [4, 9, 12, 15, 16]. It has been posited that the underlying mechanism for this resistance is that the organism behaves differently in vitro as compared to in vivo, although this has yet to be confirmed [9]. The mechanism by which the organism is also seemingly resistant to surgical washout is also currently unknown. Nonetheless, this only further emphasizes the importance of elucidating a method for obtaining accurate antimicrobial sensitivity data Table 1.

**Table 1:** Cases of postoperative endophthalmitis caused by *Sphingomonas paucimobilis*

Author, year	Age, sex	Time to presentation	Recurrence	Treatment	Initial VA	Outcome
Adams, 2006 [9]	73, female	< 24 h post op	7 months post initial endophthalmitis	Vitrectomy and posterior capsulotomy; intravitreal ceftazidime, vancomycin and dexamethasone; oral ciprofloxacin, topical prednisolone, chloramphenicol and gentamicin		Final BCVA 6/6
Seo, 2008 [12]	62, male	3 months post op	Nil	Vitrectomy, lens removal, intravitreal ceftazidime		Final BCVA 20/300
Garrido, 2014 [10]	67, female	Day 3 post op	Nil	Intravitreal vancomycin and ceftazidime; topical dexamethasone; intravenous ceftazidime		Evisceration
Huang, 2015 [14]						
Kelkar, 2016 [11]	-	Day 1 post op	-	-		Final BCVA 20/200
Mitra, 2018 [13]	16, male	1 month post op	Nil	Intravitreal vancomycin, ceftazidime and dexamethasone; oral ciprofloxacin; topical moxifloxacin and prednisolone		Final BCVA 20/60
Agarwal, 2019 [4]	17, male	Day 2 post op	Nil	3x vitrectomies; 3x courses of ceftazidime and gentamycin		Non-responsive to treatment, phthisis bulbi, NPL

In conclusion, our case report and literature review highlights the unpredictable manner in which *S. paucimobilis* endophthalmitis may initially present and behave throughout its clinical course. Given the lack of early clinical signs of residual infection, ophthalmologists may wish to consider more frequent and longer review windows for operations that are complicated by a confirmed *S. paucimobilis* infection. Further investigation is required to determine a method by which the organism’s in vivo sensitivities can be confirmed, or failing that, countermeasures developed against its resistance to standard endophthalmitis treatment.

## Data Availability

The data used during the current study are available from the corresponding author on reasonable request.

## References

[1] Vaziri K, Schwartz SG, Kishor K, Flynn HW (2015). Jr. Endophthalmitis: state of the art. Clin Ophthalmol.

[2] Pathengay A, Flynn HW, Isom RF, Miller D (2012). Endophthalmitis outbreaks following cataract surgery: causative organisms, etiologies, and visual acuity outcomes. J Cataract Refractive Surg.

[3] Stevenson LJ, Dawkins RCH, Sheorey H, McGuinness MB, Hurley AH, Allen PJ (2020). Gram-negative endophthalmitis: a prospective study examining the microbiology, clinical associations and visual outcomes following infection. Clin Exp Ophthalmol.

[4] Agarwal R, Gagrani M, Mahajan A, Sharma N (2019). Fulminant Sphingomonas paucimobilis keratitis: case study and review of literature. BMJ Case Rep.

[5] Taban M, Behrens A, Newcomb RL, Nobe MY, Saedi G, Sweet PM (2005). Acute Endophthalmitis following cataract surgery: a systematic review of the literature. Arch Ophthalmol.

[6] Lin J-N, Lai C-H, Chen Y-H, Lin H-L, Huang C-K, Chen W-F (2010). Sphingomonas paucimobilis Bacteremia in humans: 16 case reports and a literature review. J Microbiol Immunol Infect.

[7] Ryan MP, Adley CC (2010). Sphingomonas paucimobilis: a persistent Gram-negative nosocomial infectious organism. J Hosp Infect.

[8] Zhang Y, Liu ZR, Chen H, Dong WJ, Fan YC, Yu H (2012). Comparative study of bacterial status from conjunctival sac of the elder Qiang minority and Han people with dry eye in Sichuan, China. Int J Ophthalmol.

[9] Adams WE, Habib M, Berrington A, Koerner R, Steel DH (2006). Postoperative endophthalmitis caused by Sphingomonas paucimobilis. J Cataract Refract Surg.

[10] Mauri Garrido O, Borges Mendoza EB, Ramos López M, Valle Rodríguez L (2014). Escobar Román R. Endoftalmitis poscirugía de catarata por Sphingomonas paucimobilis. Rev Cubana Oftalmol.

[11] Kelkar AS, Kelkar JA, Barve PM, Mulay A, Sharma S, Amoaku W (2016). Post-clear corneal phacoemulsification endophthalmitis: profile and management outcomes at a tertiary eye care center in western India. J Ophthalmic Inflamm Infect.

[12] Seo SW, Chung IY, Kim E, Park JM (2008). A case of postoperative Sphingomonas paucimobilis endophthalmitis after cataract extraction. Korean J Ophthalmol.

[13] Mitra S, Padhi TR, Basu S, Priyadarshini SR, Sharma S (2018). Unusual microbiological presentations in polymicrobial post-operative endophthalmitis and their clinical correlations. Int Ophthalmol.

[14] Huang Y, Zhan Y, Xie L (2015). [Clinical observations of acute-onset endophthalmitis after clear corneal phacoemulsification]. Zhonghua Yan Ke Za Zhi.

[15] Ebru E, ÖZCAN A, ŞİMŞEK F, ÜNDAR İ (2015). Postpartum endogenous panophthalmitis caused by Sphingomonas paucimobilis. Cukurova Med J.

[16] Kriet M, Bouya Y, Louaya S (2011) Endogenous postpartum panophthalmitis induced by sphingomonas paucimobili. Bulletin de la Societe Belge D’ophtalmologie. (318):37–40 22003763

