# Correction: Recurrent post-operative endophthalmitis caused by Sphingomonas paucimobilis despite vitrectomy – a case and review of the literature

**DOI:** 10.1186/s12348-023-00346-1

**Published:** 2023-05-01

**Authors:** Luke Tran, Rylan Hayes, Andrew Apel, Thomas P Moloney

**Affiliations:** 1 Department of Ophthalmology, Princess Alexandra Hospital, Brisbane, QLD Australia; 2 School of Medicine, University of Queensland, Brisbane, QLD Australia; 3 The Eye Health Centre, Wickham Terrace, Brisbane, QLD Australia; 4 Vitreoretinal Unit, Department of Ophthalmology, Royal Brisbane and Women’s Hospital, Brisbane, QLD Australia

**Correction: Journal of Ophthalmic Inflammation and Infection 13, 7 (2023)**

**https://doi.org/10.1186/s12348-023-00325-6**

Following publication of the original article [1], it came to the authors' attention that the name of the author Andrew Apel had been erroneously given as 'Andrew Apel Franzco'. The published article has since been corrected and the corrected author list may be seen in this erratum. The authors thank you for reading this erratum.
